# Low-Molecular-Weight Heparin and Unfractionated Heparin Decrease Th-1, 2, and 17 Expressions

**DOI:** 10.1371/journal.pone.0109996

**Published:** 2014-11-03

**Authors:** Jing-Ning Huang, Ming-Chin Tsai, Shun-Lung Fang, Margaret Dah-Tsyr Chang, Yu-Rou Wu, Jaw-Ji Tsai, Lin-Shien Fu, Heng-Kuei Lin, Yi-Jun Chen, Tsai-Wei Li

**Affiliations:** 1 Pediatric Department, Taichung Veterans General Hospital, Taichung, Taiwan, Republic of China; 2 Medical Research, Taichung Veterans General Hospital, Taichung, Taiwan, Republic of China; 3 Department of Pediatrics, National Yang-Ming Medical University, Taipei, Taiwan, Republic of China; 4 Institute of Molecular and Cellular Biology, National Tsing Hua University, Hsinchu, Taiwan, Republic of China; 5 Department of Medical Science, National Tsing Hua University, Hsinchu, Taiwan, Republic of China; Northwestern University Feinberg School of Medicine, United States of America

## Abstract

**Background:**

We evaluated the effects of T helper cell differentiation in a mite-allergic animal model treated with inhaled heparins of different molecular weight.

**Method:**

BALB/c mice were divided into four groups: 1. Control, 2. Mite intratracheal (mIT), 3. Inhaled heparin (hIN), 4. Inhaled low-molecular-weight heparin (lmwhIN). Groups 2, 3, and 4 were sensitized twice with Der p allergen subcutaneously on day 1 and day 8. Der p allergen was administered intratracheally on day 15. Groups 3 and 4 were treated with heparin or low-molecular-weight (lmw) heparin intranasally from day 1 to 22. Splenocytes from sacrificed mice stimulated with 16 µg/ml of Der p were cultured for 72 hours. Supernatants of splenocyte were collected to analyze the effect of Interleukin (IL)17-A/F, Interferon(IFN)-γ, IL-4, IL-13, and IL-10. Serum was also collected for Der P-specific IgE level on day 23. Total RNA was extracted from spleen tissue for mRNA expression. Gene expression of Foxp3, IL-10 IFN-γ, GATA3, IL-5, and RORγt were analyzed.

**Results:**

Both hIN and lmwhIN groups had lower serum IgE level than that of the mIT group (both p<0.0001). Both hIN and lmwhIN groups showed significantly decreased transcripts of GATA-3, IFN-γ, IL-5, and RORγt mRNA in their spleen. Regarding the supernatant of splenocyte culture stimulated with Der p, compared with the mIT group, there were significant decreases in IL-17A/F, IFN-γ, IL-4, IL-13, and IL-10 secretion in inhaled hIN and lmwhIN groups.

**Conclusions:**

From this balb/c mice study, the analyses of mRNA and cytokines revealed that both intranasal heparin and lmw heparin treatment decreased the expression of Th1, Th2, and Th17 in spleen. The underlying mechanism(s) warrant further studies.

## Introduction

Bronchial asthma, a chronic inflammatory disease presented as airway obstruction, inflammatory cells infiltration, and bronchial hyper-responsiveness. T cell lymphocyte and other immune cells producing pro-inflammatory cytokines such as interleukin (IL)-4, IL-5 [1], and IL-13 [2] lead to the inflammatory response.

Heparin, a highly sulfated glycosaminoglycan (GAG), has multiple biologic activities. In addition to its well-known properties, such as its role as an anti-coagulant, heparin has also been demonstrated to have anti-inflammatory effects [3]. Previous studies have shown inhaled heparin prevents the bronchoconstrictor response to exercise [4], [5]. In addition, several studies showed biologic actions of heparin are molecular weight-dependent [6], [7]. The anti-allergic activity of heparin fractions shows an inverse relationship to the molecular weight [8]. Enoxaparin, a low-molecular-weight (lmw) heparin, is an anticoagulant used to prevent and treat deep vein thrombosis or pulmonary embolism. Previous studies report that low-molecular-weight heparin also possesses anti-inflammatory properties. LMW heparin can prevent exercise and allergen-induced bronchoconstriction [9].

Although previous studies have shown the anti-inflammatory effects of heparin and lmw heparin, there are few data on medium- to long-term inhalation treatment for asthma. Our group has demonstrated that heparin and low-molecular-weight heparin both attenuate mite-induced airway inflammation in BALB/c mice [10]. We found heparin decreased INF-γ, IL-13, IL-5, eotaxin, and IL-17A/F content in lung protein extract, and serum Der p-specific IgE level. The heparin treated groups did not reveal any adverse effect checked grossly and microscopically [10]. In the present study, we investigated the immunomodulatory effects of lmw heparin as well as heparin on Th1, Th2, and Th17 levels.

## Materials and Methods

### Animal preparation

A total 50 male BALB/c mice (6–8 weeks of age), weighing 25–30gram, were purchased from the National Laboratory Animal Center, Nangang, Taipei, Taiwan. There were 4 to 5 mice in each plastic cage, maintained at the room temperature 22±2°C with 12 hour light/dark cycle and free access to pellet food and water.

### Study protocol

**Figure 1:**
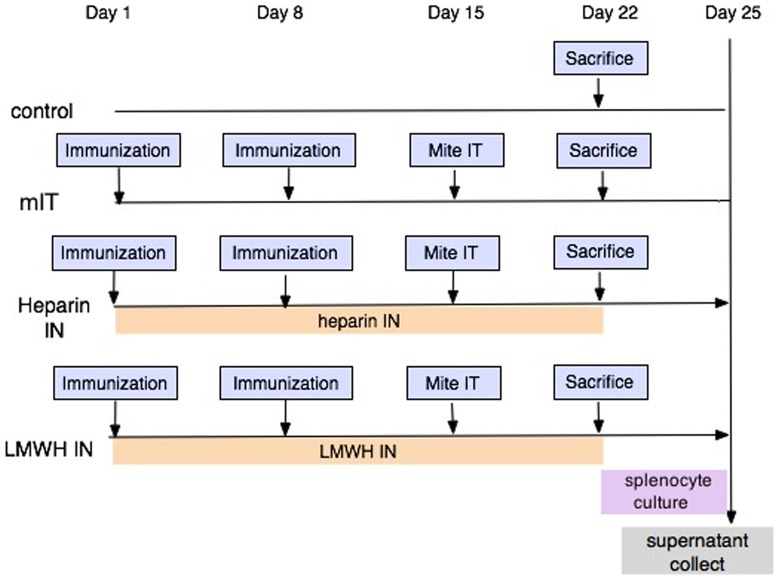
The animal protocol of this study. MIT group, heparin IN (hIN) group and low-molecular-weight heparin IN (lmwhIN) group were sensitized twice with Der p allergen subcutaneously on day 1 and day 8. Der p allergen was administered intratracheally on day 15. The hIN and lmwhIN groups were treated with heparin or lmw heparin intranasally from days 1 to 22. Splenocytes from sacrificed mice stimulated with Der p 16 ug/ml were cultured for 72 hours.

BALB/c mice were randomly divided into four groups in 3 independent repeats: 1. Control (total number N = 12), 2. Mite intratracheal (mIT, N = 12), 3. Inhaled heparin (hIN, N = 13), 4. Inhaled Low-molecular-weight heparin (lmwhIN, N = 13). Groups 2, 3, and 4 were sensitized twice with Der p allergen subcutaneously on day 1 and day 8. Der p allergen was administered intratracheally on day 15. Groups 3 and 4 were treated with heparin for 22 days. On day 23, mice were sacrificed. One fourth of the spleen was used for mRNA study. Splenocytes stimulated with Der p 16 ug/ml were cultured for 72 hours. (Figure 1) All animal work was conducted according to the relevant national and international guidelines. The protocol was approved by the Institutional Animal Care and Use Committee, Taichung Veteran General Hospital(Approval No. La-1011048). Intra-tracheal injections were performed under Isoflurane inhalation anesthesia, and sacrifice was performed by CO_2_ inhalation in a close glass chamber. All efforts were made to minimize suffering.

### Mite protein preparation

Der p was purchased from Greer Lab (Lenior, NC, USA). We extracted mite protein from the crude with phosphate buffered saline (PBS) using a glass homogenizer (Kontes Glass Company, Vineland, NJ, USA). The concentration of mite protein was then determined using the Bradford procedure (Bio-Rad protein Assay; Bio-Rad, Hercules, CA, USA).

### Sensitization

Mite crude extract allergen (1.6 mg/mL) was well-emulsified in complete Freund's adjuvant (CFA; Sigma, St Louis, MO, USA) at a ratio of 1∶1 at 4°C. The mIT, hIN, and lmwhIN groups were immunized subcutaneously with 50 microliters of emulsified mite protein on day 1 and day 8.

### Intratracheal administration of mite protein

Ten micro-liter of mite crude extract (2 mg/mL), dissolved in PBS, was administered to each mouse intratracheally on day 15.

### Intranasal administration of heparin and low-molecular-weight heparin

Heparin was purchased from China Chemical and Pharmaceutical Company (Taipei, Taiwan). Each ml contained 5000IU heparin. LMW heparin (lmwh) was purchased from Sanofi Winthrop Industry (Maisons-Alfort, France). Each ml contained lmw heparin 100 mg, and the activity was equivalent to 10,000 anti-Xa IU. Lmw Heparin was dissolved in 4% Glucose water to the final concentration of 0.0015 mg/uL (0.15 IU/uL). Ten micro-liters of heparin or lmwh heparin were then administered to each mouse intranasally from day 1 to day 22.

### Measurement of Der p-specific IgE antibody

All Balb/c mice were sacrificed, and the blood was obtained from the inferior vena cava on day 23. Serum Der p-specific IgE level was measured by enzyme-linked immunosorbent assay (ELISA). The micro-plates were coated with mite crude extract allergen (50 mg/ml) and cultured overnight at 4°C. The plates were blocked with 10% fetal bovine serum (FBS) in PBS at room temperature for 2 hours. After washing with PBS containing 0.05% Tween 20 (PBST), diluted serum samples (1/10 dilution) were added to the wells of the micro-plates and incubated at 4°C overnight. After washing with PBST, the plates were incubated with rat anti-mouse IgE biotinylated mono- clonal antibody (specific for mouse IgE-heavy chain; Abcam) at room temperature for 2 hours. After the second wash with PBST, the wells were incubated with horseradish-peroxidase (HRP) streptavidin conjugate (Zymed for IgE) at room temperature for 1 hour. The wells were washed and then incubated with TMB substrate (BD Bioscience) in the dark, at room temperature for 30 minutes. The enzyme reaction was stopped by adding 2N H2SO4, and the absorption at 450 nm was measured using an ELISA reader.

### Splenocyte preparation

Spleen was aseptically removed from mice. Single cell suspensions were obtained by mincing the spleen and gently pressing the fragments through a 70 µm stainless steel mesh. The resultant suspension was left to stand for 15 minutes to separate large debris. The suspension was mixed with RBC lysis for 5 minutes at room temperature. The splenocytes were resuspended in RPMI 1640 (GIBCO) containing 10% fetal bovine serum (FBS, Hyclone), 100U/mL penicillin, and 100 µg/ml streptomycin. Cell concentration was adjusted to 5×10^6^ cells/mL using RPMI 1640 medium.

### Splenocyte incubation

Using sterile 96-well culture plates, 5×10^6^ cells/mL were seeded in 200 µl of medium. Splenocytes were incubated for 72 hours with 16u g/ml of Der p in CO2.

### Cytokine analysis of splenocyte supernatants by ELISA

The cytokine levels of IL-4, IL-10, and IFN-γ of the splenocyte culture supernatants were examined using BD OptEIATM Set Mouse IL-4, IL-10, and IFN-γ kits (BD Bioscience, San Jose, CA, USA). The cytokine protein levels of IL-13 and IL-17 A/F were examined using R&D DuoSet ELISA Development system mouse IL-13 and IL-17 kits (R&D Systems, Minnesota, USA). The kits were used according to the manufacturer's instructions. The micro-plates used in these assays were read at 450 nm and 520 nm with an ELISA reader (Thermo Labsystems, Waltham, MA, USA).

### Quantitative RT-PCR (qRT-PCR) analysis of mRNA transcripts

Spleen tissue was submerged in RNAlater RNA stabilization Reagent (QIAGEN, USA) following the manufacturer's protocol. Total RNA was extracted from spleen tissue with TRIzol reagent (Invitrogen, USA). The purity of RNA was acceptable when the OD at 260 and 280 nm (A260/280) was between 1.8 and 2.0. Total RNA(5ug) was reverse-transcribed using High-Capacity cDNA Reverse Transcription kits (Cat. 4368814)(Applied Biosystems) and random primers (Applied Biosystems). Quantitative PCR was performed by StepOne(tm) Real-Time PCR System (Applied Biosystems) with SYBR Green PCR Master Mix (Roche), and 1 ug cDNA was added to a volume of 20 uL. Primer sequences were as follows: GATA-3, 5′- CGACCCCTTCTACTTGCGTT-3′ and 5′- TGGAATGCAGACACCACCTC-3′; IFN-γ 5′- GCTCTTCCTCATGGCTGTTT-3′ and 5′- GTCACCATCCTTTTGCCAGT-3′; IL-5, 5′- GAAGTGTGGCGAGGAGAGAC-3′ and 5′- GCACAGTTTTGTGGGGTTTT-3′; RORγt, 5′- GCTCTGCCAGAATGACCAGA-3′ and 5′- CAGCTCCACACCACCGTATT-3′ [11]

### Statistical analysis

Data were expressed as the mean ± standard deviation (SD). Analysis was performed with Mann-Whitney U-test for comparison of two groups. Differences with a p value <0.05 were considered significant. Analysis was performed using the Statistical Package for the Social Sciences (Version 10.1, SPSS, Chicago, IL, USA)

## Results

### Der. P specific serum IgE level

**Figure 2:**
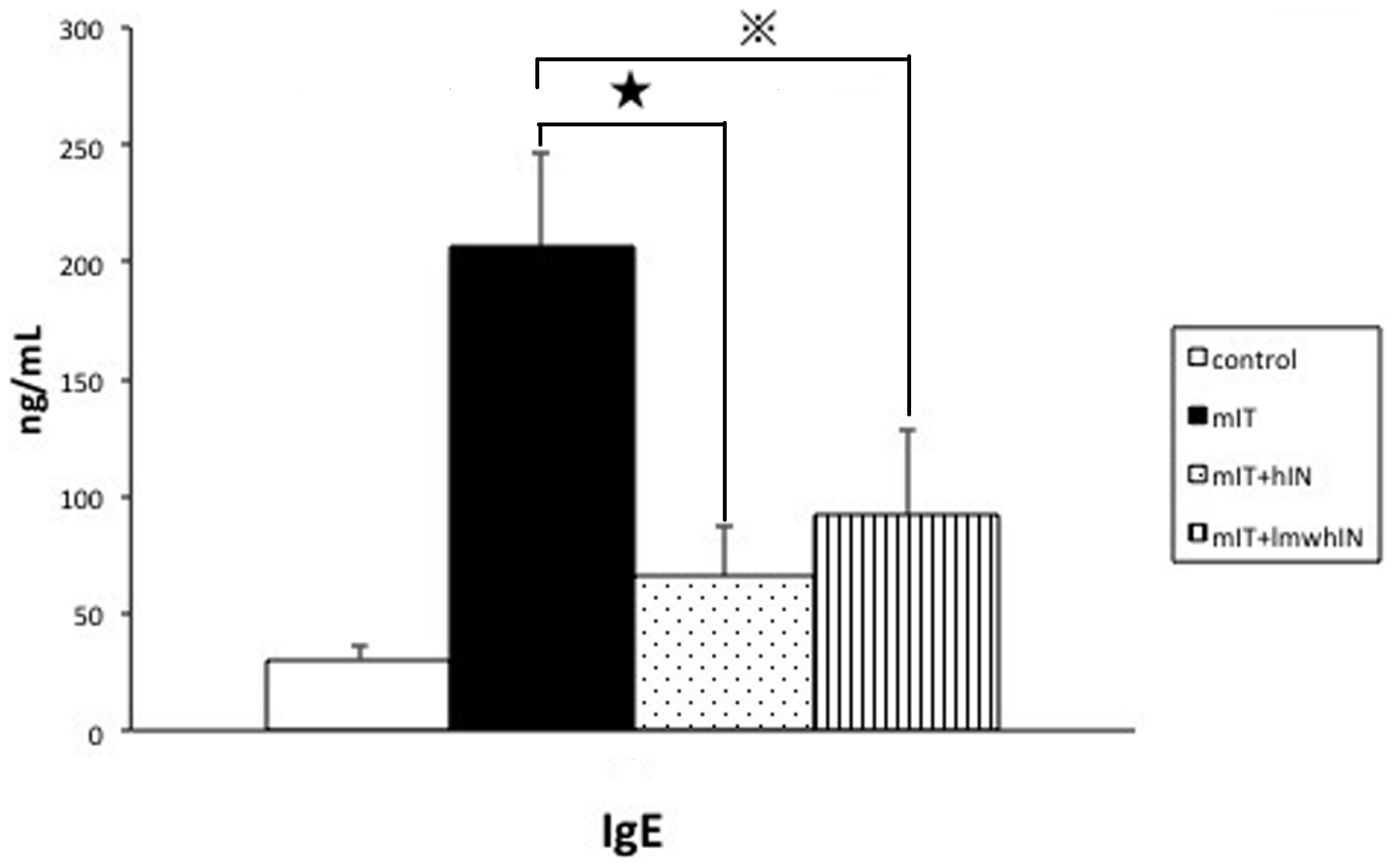
Serum Der p-specific IgE levels. Serum Der p-specific IgE levels were significantly decreased in hIN and lmwhIN groups. Control group, mIT group, hIN group, and lmwhIN group were analyzed. ^★^p<0.00000001, ^
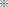
^p<0.000001.

To analyze the inhibitory effect of heparin and lmw heparin on mite sensitization, we measured Der P specific serum IgE level in all four groups. The mIT group had a higher IgE level than that of the control group (mIT vs. control as 206.21±38.03 vs. 30.50±6.22 ng/mL). Furthermore, both the heparin group and lmw heparin group showed a significantly lower level of IgE compared to that of the mIT group (hIN and lmwhIN vs. mIT as 65.42±20.80, and 92.22±34.67 vs 206.21±38.03 ng/mL, both p<0.000001). (Figure 2)

### Decreased mRNA expression in heparin and low-molecular-weight heparin treatment groups

**Figure 3:**
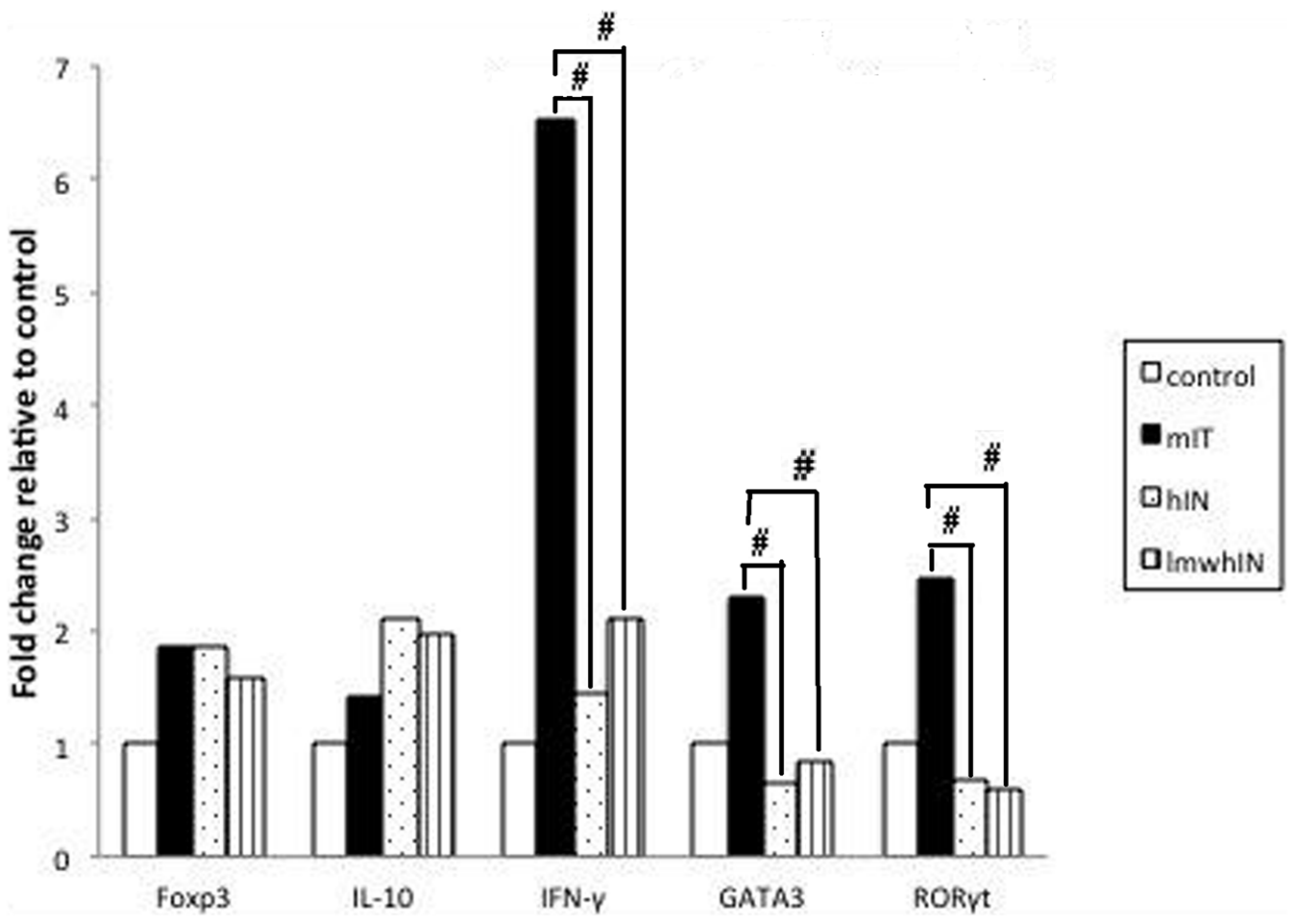
Spleen mRNA expression. Spleen mRNA expression in Foxp3, IL-10, IFN-γ, GATA-3, and RORγt were decreased in hIN and lmwhIN groups. #p = 0.05.

To evaluate differences in the relative mRNA expression in Treg, Th1, Th2 and Th17 in the heparin and lmwh groups, we analyzed spleen from 4 mice in each group mRNA including Foxp3, IL-10, GATA-3, IFN-γ, IL-5, and RORγt by qRT-PCR. We found that both hIN and lmwhIN groups had significant lower transcripts in GATA-3, IFN-γ, IL-5, and RORγt mRNA (p = 0.05). (Figure 3)

### Cytokine levels in supernatant from splenocyte culture

**Figure 4:**
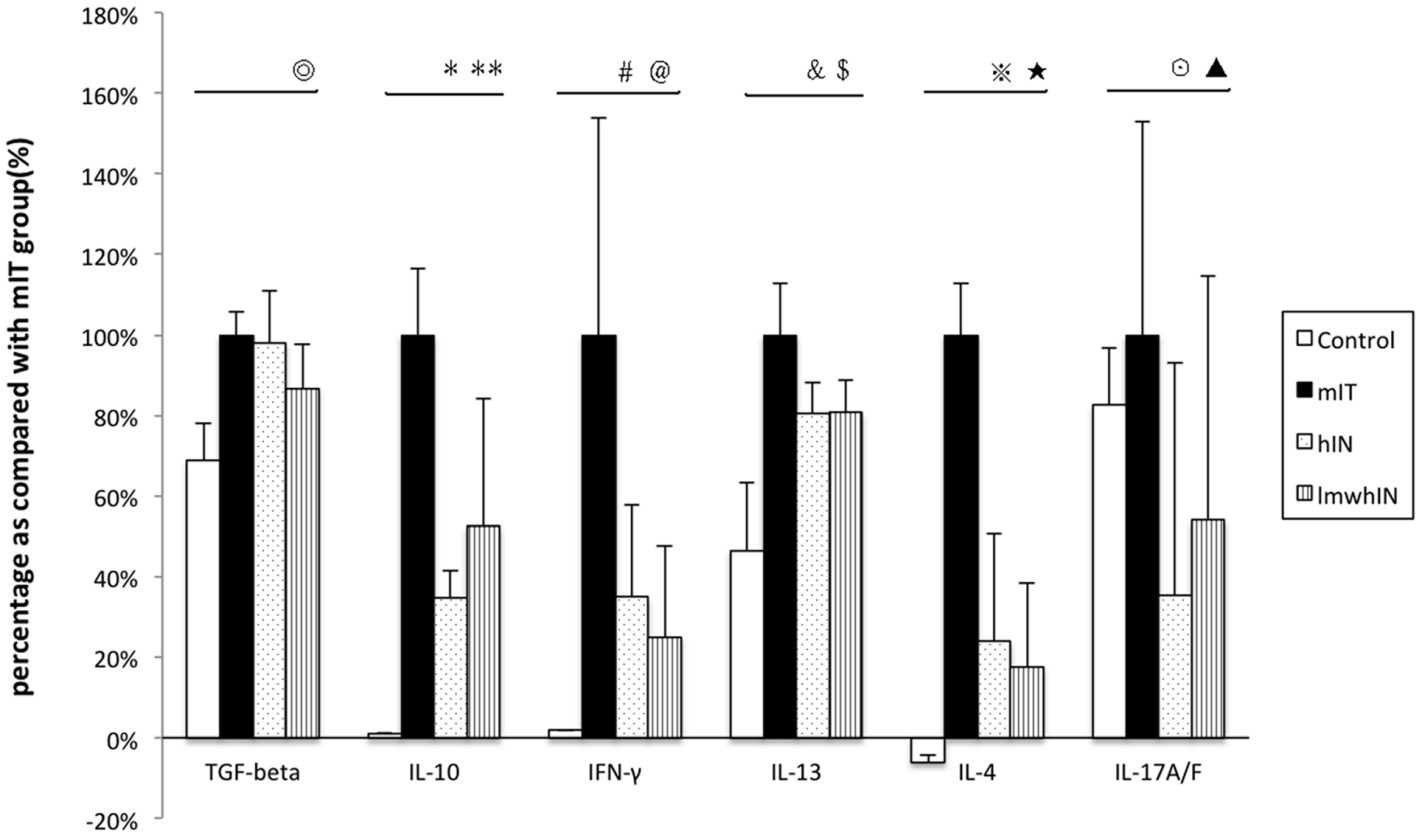
Cytokine levels in supernatants from splenocyte culture stimulated with mite. Cytokine levels in splenocyte supernatants are shown in this figure. Due to the large differences between cytokines level, we showed each cytokine level as a percentage of the corresponding level in the mIT group. The levels of mIT group are as follows: TGF-beta 2448.25±136.31 pg/mL, IL-10 5.93±0.98 pg/mL, IFN-γ 16.27±8.80 ng/mL, IL-13 2102.10±269.55 pg/mL, IL-4 159.40±20.51 pg/mL, IL-17A/F 708.6±374.52 pg/mL. ^⊚^p = 0.012, *p = 0.005, **p = 0.013, ^#^p = 0.034, ^@^p = 0.0038, ^&^p = 0.037,^ $^p = 0.033, p = 0.001, ^★^p = 0.001, ^⊙^p = 0.005, ^▴^p = 0.034.

We measured the cytokine secretion from cultured splenocyte in all study animals. Both heparin and low molecular heparin groups revealed significantly lower IL-17A/F, IFN-γ, IL-4, IL-13, and IL-10 levels as compared to those of the mIT group (Figure 4). The results were as follows: for IL-17 A/F level, the values in the hIN group and lmwhIN group were 249.90±409.09 and 383.36±428.56 pg/mL, respectively, compared with the mIT group, which had a value of 708.6±374.52 pg/mL, p = 0.005, 0.013. For Interferon-γ level, values in the hIN group and lmwhIN group were 5681.48±3327.01 and 4058.77±3371.03 pg/mL, respectively, as compared with the value in the mIT group 16269.26±7615.62 pg/mL, p = 0.037, 0.033. With regard to IL-4 level, the hIN group and lmwhIN group had values of 38.15±42.78 and 27.90±33.36 pg/mL, respectively, compared with 159.40±20.51 pg/mL, p = 0.034, 0.0038 in the mIT group. For IL-13 level, the values of the hIN group and lmwhIN group were 1690.42±160.48 and 1697±19±169.88 pg/mL, respectively, compared with the mIT group, which had a value of 2102.10±269.55 pg/mL, both p = 0.001. For IL-10 level, the hIN group and lmwhIN group had values of 2061.04±396.23 and 3112.60±1872.08 pg/mL, respectively, compared with the mIT group, which had a value of 5931.88±975.99 pg/mL, p = 0.05, 0.034.

## Discussion

In this study, the results demonstrated that intranasal heparin administration modulated the expressions in Th1, 2, and 17 in the spleen. There was increased mRNA transcripts in INF-γ, GATA-3, and ROR-γT in the spleen of mIT mice on the day of sacrifice, while those treated with heparin of different molecular sizes via intranasal route had decreased mRNA expression in the aforementioned factors. This indicates that intranasal heparin administration exerts a systemic effect in addition to the local effect on airway and lung, as we demonstrated before [10]. The cultured splenocyte stimulated with Der p 16ug/ml for 72 hours also revealed increased INF-γ, IL-4, IL-13, and IL17A/F in the mIT group and the splenocytes from the heparin IN-treated groups had much lower secretions in these cytokines.

The animal model in the present study resulted in increased expression of Th1 and Th17, in addition to Th2. Asthma has long been thought to be related to Th2 deviation. In recent years, increasing evidence has shown the roles of Th1 [12] [13] [14] [15] and Th17 [15] [16] [17] [18], in addition to Th2 cells, in the pathogenesis of asthma.

However, to date, most murine-based studies on asthma used ovalbumin (OVA) to sensitize, then inhalation challenge to establish the airway allergic inflammation. The OVA model was used to study Th2 activation in many previous studies. In recent years, it was also demonstrated to enhance Th17 expression [19] [20]. The effects may be different from those seen in the real world, in which mite is the major allergen that induces asthma. In recent years, an increasing number of murine asthma studies have used mite as the allergen. Some studies have shown that mite sensitization followed by inhalation activated Th1 and Th2 [21], though others did not [22] [23]. Lan et al. sensitized the mice with OVA plus alum, then administered mite inhalation to the mice (OVA + mite) [22]. Compared with the mice treated with OVA inhalation, the OVA + mite group had higher IL-17 and IL-23 than that of the OVA group. For IL-5, the OVA + mite group had an even higher level of BALF and serum than that of the OVA group. INF-γ did not increase in the OVA + mite group. Taken together, these data show that mite exposure increased INF-γ and IL-17 production, in addition to Th2 cytokines. Our model is compatible with previous models, and INF-γ production can be further enhanced by the usage of CFA in sensitization.

Heparin, a highly acidic polymer, exhibits biologic effects that are dependent on both specific and nonspecific ionic interactions which are mediated by sequence composition, charge density, charge distribution, and molecular size. In addition to its known anticoagulant activity, heparin also has many non-coagulant properties including anti-inflammation [24] [25] [26], inhibition of complement activation [24], neutrophil aggravation and elastase release [27], as well as eosinophil influx [28] [29]. Several studies have shown short-term [4] [30], and medium-term [10] anti- inflammatory effects in airway allergy. To the best of our knowledge, this is the first study to demonstrate an immunomodulatory effect with the use of heparin.

The interactions between Th1, 2 and 17 are mutually inhibited. The present data from this study do not either support heparins deviate the differentiation of T helper cell, or increase the activity of T regulatory cell. The suppression in Th1, 2, and 17 activations imply that heparin exhibits some inhibition effects from upper stream to Th0 differentiation. Adhesion between antigen-presenting cell and T helper cell and its subsequent activation play a central role in the inflammatory response. Many reports showed that heparin can interfere with adhesion of leukocytes by inhibiting L-selectin and P-selectin binding [31] [32] or mediating cell surface expression of CD11/CD18 complexes [33]. Recently, heparin and low molecular heparin have been demonstrated to interfere the reaction between surface heparan sulfate to cell penetrating peptide [34]. With the advance of glycol-immunology, more detailed mechanism of heparins would be disclosed.

NF-κB is a major transcription factor that regulates genes involved in immunity. Through a cascade of phosphorylation events, the kinase complex is activated and NF-κB enters the nucleus to regulate genes that are involved in T cell development and proliferation [35]. Heparin can internalize into the cytoplasm and bind electrostatically to the positively charged sequence of NF-κB, to prevent the translocation of NF-κB to the nucleus [36] [37] [38] [39], which reduces inflammatory gene activation and downregulates the secretion of inflammatory cytokine, chemokine, and adhesion molecule production [3]. In this study, we have demonstrated that heparin of different sizes can downregulate the expression of transcription factors, GATA-3 and RORγt, Besides NF-κB, GATA-3, and RORγt, there are various pathways related to our results, including MAPK [40], JAK [41], and others. The effects of heparin on these pathways require further study.

Another explanation of our results is the heparin binding to several cytokines involving in T helper differentiation. Hasan M et al [42] has demonstrated the heparin binding domain on IL-12 to be the subunit p40, which is also a critical protein for Th17 differentiation [43]. Besides, heparins also bind to INF-γ, as reported by Hasan et al. The interaction of heparin and cytokine, especially the glycosaminoglycan part of the molecule, is an important issue in recent publications [44], [45]. The results of the present study provide a clue in this aspect.

In conclusion, we have demonstrated that heparin of different sizes administered via inhalation can decrease Th1, 2, and 17 expression in spleen, and in splenocytes stimulated with Der p. The underlying mechanism warrants further study.

## References

[1] Robinson DS, Hamid Q, Ying S, Tsicopoulos A, Barkans J, et al (1992) Predominant TH2-like bronchoalveolar T-lymphocyte population in atopic asthma. N Engl J Med 326: 298–304. 1530827 10.1056/NEJM199201303260504

[2] Wills-Karp M, Chiaramonte M (2003) Interleukin-13 in asthma. Curr Opin Pulm Med 9: 21–27. 12476080 10.1097/00063198-200301000-00004

[3] Young E (2008) The anti-inflammatory effects of heparin and related compounds. Thromb Res 122: 743–752. 17727922 10.1016/j.thromres.2006.10.026

[4] Ahmed T, Garrigo J, Danta I (1993) Preventing bronchoconstriction in exercise-induced asthma with inhaled heparin. N Engl J Med 329: 90–95. 8510708 10.1056/NEJM199307083290204

[5] Garrigo J, Danta I, Ahmed T (1996) Time course of the protective effect of inhaled heparin on exercise-induced asthma. Am J Respir Crit Care Med 153: 1702–1707. 8630624 10.1164/ajrccm.153.5.8630624

[6] Cifonelli JA (1974) The relationship of molecular weight, and sulfate content and distribution to anticoagulant activity of heparin preparations. Carbohydr Res 37: 145–154. 4473267 10.1016/s0008-6215(00)87070-6

[7] Laurent TC, Tengblad A, Thunberg L, Hook M, Lindahl U (1978) The molecular-weight-dependence of the anti-coagulant activity of heparin. Biochem J 175: 691–701. 743219 10.1042/bj1750691 PMC1186120

[8] Martinez-Salas J, Mendelssohn R, Abraham WM, Hsiao B, Ahmed T (1998) Inhibition of allergic airway responses by inhaled low-molecular-weight heparins: molecular-weight dependence. J Appl Physiol 84: 222–228. 9451639 10.1152/jappl.1998.84.1.222

[9] Ahmed T, Gonzalez BJ, Danta I (1999) Prevention of exercise-induced bronchoconstriction by inhaled low-molecular-weight heparin. Am J Respir Crit Care Med 160: 576–581. 10430731 10.1164/ajrccm.160.2.9812076

[10] Fu LS, Tsai JJ, Chen YJ, Lin HK, Tsai MC, et al (2013) Heparin protects Balb/c mice from mite-induced airway allergic inflammation. Int J Immunopathol Pharmacol 26: 349–359. 23755750 10.1177/039463201302600208

[11] Yang M, Yang C, Mine Y (2010) Multiple T cell epitope peptides suppress allergic responses in an egg allergy mouse model by the elicitation of forkhead box transcription factor 3- and transforming growth factor-beta-associated mechanisms. Clin Exp Allergy 40: 668–678. 20082619 10.1111/j.1365-2222.2009.03442.x

[12] Green RH, Brightling CE, Woltmann G, Parker D, Wardlaw AJ, et al (2002) Analysis of induced sputum in adults with asthma: identification of subgroup with isolated sputum neutrophilia and poor response to inhaled corticosteroids. Thorax 57: 875–879. 12324674 10.1136/thorax.57.10.875 PMC1746199

[13] Kim YK, Oh SY, Jeon SG, Park HW, Lee SY, et al (2007) Airway exposure levels of lipopolysaccharide determine type 1 versus type 2 experimental asthma. J Immunol 178: 5375–5382. 17404323 10.4049/jimmunol.178.8.5375

[14] Kim HY, DeKruyff RH, Umetsu DT (2010) The many paths to asthma: phenotype shaped by innate and adaptive immunity. Nat Immunol 11: 577–584. 20562844 10.1038/ni.1892 PMC3114595

[15] Holgate ST (2008) Pathogenesis of Asthma. Clinical & Experimental Allergy 38: 872–897. 18498538 10.1111/j.1365-2222.2008.02971.x

[16] McKinley L, Alcorn JF, Peterson A, Dupont RB, Kapadia S, et al (2008) TH17 cells mediate steroid-resistant airway inflammation and airway hyperresponsiveness in mice. J Immunol 181: 4089–4097. 18768865 10.4049/jimmunol.181.6.4089 PMC3638757

[17] Al-Ramli W, Prefontaine D, Chouiali F, Martin JG, Olivenstein R, et al (2009) T(H)17-associated cytokines (IL-17A and IL-17F) in severe asthma. J Allergy Clin Immunol 123: 1185–1187. 19361847 10.1016/j.jaci.2009.02.024

[18] Barczyk A, Pierzchala W, Sozanska E (2003) Interleukin-17 in sputum correlates with airway hyperresponsiveness to methacholine. Respir Med 97: 726–733. 12814161 10.1053/rmed.2003.1507

[19] Zhang B, An J, Shimada T, Liu S, Maeyama K (2012) Oral administration of Enterococcus faecalis FK-23 suppresses Th17 cell development and attenuates allergic airway responses in mice. Int J Mol Med 30: 248–254. 22641478 10.3892/ijmm.2012.1010

[20] Shi Y, Jin Y, Guo W, Chen L, Liu C, et al (2012) Blockage of nerve growth factor modulates T cell responses and inhibits allergic inflammation in a mouse model of asthma. Inflamm Res 61: 1369–1378. 22871964 10.1007/s00011-012-0538-3

[21] Lin LH, Zheng P, Yuen JW, Wang J, Zhou J, et al (2012) Prevention and treatment of allergic inflammation by an Fcgamma-Der f2 fusion protein in a murine model of dust mite-induced asthma. Immunol Res 52: 276–283. 22539132 10.1007/s12026-012-8339-x

[22] Kim SJ, Kim JW, Kim YH, Lee SH, Yoon HK, et al (2009) Effects of tranilast and pentoxifylline in a mouse model of chronic asthma using house dust mite antigen. J Asthma 46: 884–894. 19905913 10.3109/02770900903089998

[23] Phipps S, Lam CE, Kaiko GE, Foo SY, Collison A, et al (2009) Toll/IL-1 signaling is critical for house dust mite-specific helper T cell type 2 and type 17 [corrected] responses. Am J Respir Crit Care Med 179: 883–893. 19246719 10.1164/rccm.200806-974OC

[24] Weiler JM, Edens RE, Linhardt RJ, Kapelanski DP (1992) Heparin and modified heparin inhibit complement activation in vivo. J Immunol 148: 3210–3215. 1578145

[25] Yagmurdur MC, Colak T, Emiroglu R, Karabay G, Bilezikci B, et al (2003) Antiinflammatory action of heparin via the complement system in renal ischemia-reperfusion. Transplant Proc 35: 2566–2570. 14612020 10.1016/j.transproceed.2003.08.076

[26] Wang L, Brown JR, Varki A, Esko JD (2002) Heparin's anti-inflammatory effects require glucosamine 6-O-sulfation and are mediated by blockade of L- and P-selectins. J Clin Invest 110: 127–136. 12093896 10.1172/JCI14996 PMC151027

[27] Brown RA, Lever R, Jones NA, Page CP (2003) Effects of heparin and related molecules upon neutrophil aggregation and elastase release in vitro. Br J Pharmacol 139: 845–853. 12813008 10.1038/sj.bjp.0705291 PMC1573888

[28] Hecht I, Hershkoviz R, Shivtiel S, Lapidot T, Cohen IR, et al (2004) Heparin-disaccharide affects T cells: inhibition of NF-kappaB activation, cell migration, and modulation of intracellular signaling. J Leukoc Biol 75: 1139–1146. 15020655 10.1189/jlb.1203659

[29] Vancheri C, Mastruzzo C, Armato F, Tomaselli V, Magri S, et al (2001) Intranasal heparin reduces eosinophil recruitment after nasal allergen challenge in patients with allergic rhinitis. J Allergy Clin Immunol 108: 703–708. 11692092 10.1067/mai.2001.118785

[30] Ceyhan B, Celikel T (1995) EFfect of inhaled heparin on methacholine-induced bronchial hyperreactivity. CHEST Journal 107: 1009–1012. 10.1378/chest.107.4.1009 7705106

[31] Nelson RM, Cecconi O, Roberts WG, Aruffo A, Linhardt RJ, et al (1993) Heparin oligosaccharides bind L- and P-selectin and inhibit acute inflammation. Blood 82: 3253–3258. 7694675

[32] Asa D, Gant T, Oda Y, Brandley BK (1992) Evidence for two classes of carbohydrate binding sites on selectins. Glycobiology 2: 395–399. 1281020 10.1093/glycob/2.5.395

[33] Diamond MS, Alon R, Parkos CA, Quinn MT, Springer TA (1995) Heparin is an adhesive ligand for the leukocyte integrin Mac-1 (CD11b/CD1). J Cell Biol 130: 1473–1482. 7559767 10.1083/jcb.130.6.1473 PMC2120570

[34] Fang SL, Fan TC, Fu HW, Chen CJ, Hwang CS, et al (2013) A novel cell-penetrating peptide derived from human eosinophil cationic protein. PLoS One 8: e57318. 23469189 10.1371/journal.pone.0057318 PMC3587609

[35] Livolsi A, Busuttil V, Imbert V, Abraham RT, Peyron JF (2001) Tyrosine phosphorylation-dependent activation of NF-kappa B. Requirement for p56 LCK and ZAP-70 protein tyrosine kinases. Eur J Biochem 268: 1508–1515. 11231305 10.1046/j.1432-1327.2001.02028.x

[36] Young E, Venner T, Ribau J, Shaughnessy S, Hirsh J, et al (1999) The binding of unfractionated heparin and low molecular weight heparin to thrombin-activated human endothelial cells. Thromb Res 96: 373–381. 10605952 10.1016/s0049-3848(99)00125-5

[37] Letourneur D, Caleb BL, Castellot JJ Jr (1995) Heparin binding, internalization, and metabolism in vascular smooth muscle cells: II. Degradation and secretion in sensitive and resistant cells. J Cell Physiol 165: 687–695. 7593249 10.1002/jcp.1041650328

[38] Dudas J, Ramadori G, Knittel T, Neubauer K, Raddatz D, et al (2000) Effect of heparin and liver heparan sulphate on interaction of HepG2-derived transcription factors and their cis-acting elements: altered potential of hepatocellular carcinoma heparan sulphate. Biochem J 350 Pt 1: 245–251. 10926850 PMC1221248

[39] Akimoto H, Ito H, Tanaka M, Adachi S, Hata M, et al (1996) Heparin and heparan sulfate block angiotensin II-induced hypertrophy in cultured neonatal rat cardiomyocytes. A possible role of intrinsic heparin-like molecules in regulation of cardiomyocyte hypertrophy. Circulation 93: 810–816. 8641011 10.1161/01.cir.93.4.810

[40] Krstic A, Kocic J, Ilic V, Mojsilovic S, Okic-Dordevic I, et al (2012) Effects of IL-17 on erythroid progenitors growth: involvement of MAPKs and GATA transcription factors. J Biol Regul Homeost Agents 26: 641–652. 23241114

[41] Coskun M, Salem M, Pedersen J, Nielsen OH (2013) Involvement of JAK/STAT signaling in the pathogenesis of inflammatory bowel disease. Pharmacol Res 76C: 1–8. 10.1016/j.phrs.2013.06.007 23827161

[42] Hasan M, Najjam S, Gordon MY, Gibbs RV, Rider CC (1999) IL-12 is a heparin-binding cytokine. J Immunol 162: 1064–1070. 9916734

[43] Jones SA, Sutton CE, Cua D, Mills KH (2012) Therapeutic potential of targeting IL-17. Nat Immunol 13: 1022–1025. 23080193 10.1038/ni.2450

[44] Peysselon F, Ricard-Blum S (2013) Heparin-protein interactions: From affinity and kinetics to biological roles. Application to an interaction network regulating angiogenesis. Matrix Biol. 10.1016/j.matbio.2013.11.001 24246365

[45] Harris N, Kogan FY, Il'kova G, Juhas S, Lahmy O, et al (2014) Small molecule inhibitors of protein interaction with glycosaminoglycans (SMIGs), a novel class of bioactive agents with anti-inflammatory properties. Biochim Biophys Acta 1840: 245–254. 24060749 10.1016/j.bbagen.2013.09.023

